# Bio-Guided Fractionation and Molecular Networking Reveal Fatty Acids to Be Principal Anti-Parasitic Compounds in Nordic Seaweeds

**DOI:** 10.3389/fphar.2021.674520

**Published:** 2021-06-02

**Authors:** Charlotte Smith Bonde, Louis Bornancin, Yi Lu, Henrik Toft Simonsen, María Martínez-Valladares, Miguel Peña-Espinoza, Helena Mejer, Andrew R. Williams, Stig Milan Thamsborg

**Affiliations:** ^1^ Department of Veterinary and Animal Sciences, University of Copenhagen, Frederiksberg, Denmark; ^2^ Department of Biotechnology and Biomedicine, Technical University of Denmark, Lyngby, Denmark; ^3^ Instituto de Ganadería de Montaña (CSIC-Universidad de León), Department of Animal Health, León, Spain; ^4^ Instituto de Farmacologia y Morfofisiologia, Facultad de Ciencias Veterinarias, Universidad Austral de Chile, Valdivia, Chile

**Keywords:** macroalgae, fatty acids, anthelminthic, stearidonic acid, eicosapentaenoic acid, alpha-linolenic acid

## Abstract

Widespread use of antimicrobial drugs has led to high levels of drug-resistance in pathogen populations and a need for novel sources of anti-bacterial and anti-parasitic compounds. Macroalgae (seaweed) are potentially a rich source of bioactive compounds, and several species have traditionally been used as vermifuges. Here, we investigated the anti-parasitic properties of four common cold-water Nordic seaweeds; *Palmaria palmata* (Rhodophyta), *Laminaria digitata*, *Saccharina latissima* and *Ascophyllum nodosum* (Ochrophyta, Phaeophyceae). Screening of organic extracts against helminths of swine (*Ascaris suum*) and sheep (*Teladorsagia circumcincta*) revealed that *S. latissima* and *L. digitata* had particularly high biological activity. A combination of molecular networking and bio-guided fractionation led to the isolation of six compounds from extracts of these two species identified in both fermented and non-fermented samples. The six isolated compounds were tentatively identified by using MS-FINDER as five fatty acids and one monoglyceride: Stearidonic acid (**1**), Eicosapentaenoic acid (**2**), Alpha-Linolenic acid (**3**), Docosahexaenoic acid (**4**), Arachidonic acid (**5**), and Monoacylglycerol (MG 20:5) (**6**). Individual compounds showed only modest activity against *A. suum*, but a clear synergistic effect was apparent when selected compounds were tested in combination. Collectively, our data reveal that fatty acids may have a previously unappreciated role as natural anti-parasitic compounds, which suggests that seaweed products may represent a viable option for control of intestinal helminth infections.

## Introduction

Gastrointestinal (GI) parasitic helminths are major pathogens causing reduced health, welfare and productivity in livestock (Fitzpatrick, 2013). Due to ever-increasing drug resistance in livestock helminth populations (Kaplan and Vidyashankar, 2012; Sangster et al., 2018), it is imperative to identify alternative treatments to the available commercial drugs. Natural products have been used for centuries for different medicinal purposes, including treatment of parasitic infections (Waller et al., 2001; Athanasiadou et al., 2007) and for most small scale farmers in resource-poor areas these are still the main source of medicine (Githiori et al., 2005). Several studies of bioactive forages against helminths have shown promising results (Butter et al., 2001; Hoste et al., 2015; Mueller-Harvey et al., 2019), but few studies have examined plants and algae of marine origin.

Macroalgae or seaweeds are very diverse and abundant and their high levels of bioactive compounds could hold vast opportunities for the pharmaceutical industry and medicinal exploitation (Faulkner, 1999). Most research in bioactive compounds of marine origin has been on their anti-cancer properties (Hu et al., 2015) while other seaweed compounds have positive effects on gut health, e.g. antimicrobial, anti-inflammatory and anti-parasitic properties (Holdt and Kraan, 2011; Mayer et al., 2017). The Asian seaweed, *Digenea simplex* (Rhodophyta) has for centuries been used as a deworming agent against the human nematode *Ascaris lumbricoides*, due to the active compound kainic acid (Schwimmer and Schwimmer, 1955). The Australian seaweed *Notheia anomala* (Ochrophyta, Phaeophyceae) has also been proven to have *in vitro* nematocidal effects against two ovine GI nematodes, *Haemonchus contortus* and *Trichostrongylus colubriformis*, with the active compounds found to be tetrahydrofurans (Capon et al., 1998), and organic compounds isolated from brown macroalgae such as 6-tridecylsalisalicyclic acid have been shown to induce subcuticular tissue damage in adult *H. contortus* and also changes structural features of L4s (Taki et al., 2020). We therefore investigated local seaweeds common in Nordic waters for their anti-parasitic properties targeting helminths.

In the present study, we examined the anti-parasitic properties of three species of brown macroalgae (*Laminaria digitata*, *Saccharina latissima* and *Ascophyllum nodosum*) and one red macroalgae (*Palmaria palmata*), which are common in Nordic waters and have been used for centuries as occasional livestock feed when resources were scarce (Makkar et al., 2016). We prepared crude extracts from the four species and tested the nematocidal activity of the extracts against the pig nematode *A. suum*, a major parasite of pigs worldwide (Dold and Holland, 2011; Sanchez-Vazquez et al., 2012), and the sheep nematode *Teladorsagia circumcincta*. We included extracts of lactobacilli-fermented and non-fermented samples of the same origin, since fermentation is believed to increase digestibility of feed components (Canibe and Jensen, 2012; Yuan et al., 2017) and enhance the bioavailability of natural products (Hussain et al., 2016). The most active extracts were fractionated in order to identify the biologically active compounds.

## Results and Discussion

The seaweeds (*S. latissima, A. nodosum, L. digitata*, *P. palmata*) were selected based on their commercial availability and expected low toxicity, and subsequently harvested from Nordic waters (Table 1). We prepared hexane, dichloromethane:methanol (DCM:MeOH) and methanol:water (MeOH:H_2_O) crude extracts of both fermented (FER) and non-fermented (non-FER) seaweeds. The activity of the extracts was then assessed against third stage larvae (L3) from *A. suum*. After 24 h exposure, hexane extracts of non-fermented *S. latissima* and *P. palmaria*, and DCM:MeOH extracts of non-fermented *S. latissima* and *L. digitata* induced a larval mortality of >90%. Hexane and DCM:MeOH extracts from fermented *S. latissima* and the DCM:MeOH extract of fermented *L. digitata* also induced more than 90% mortality (Table 1). Over a period of 72 h, the mortality of L3 exposed to the extracts increased for almost all seaweed extracts (Supplementary material Image 1). A difference was observed between MeOH:H_2_O extracts of *S. latissima* non-fermented samples of the two different locations (*p* = 0.009), indicating higher activity of polar compounds in the extract from the Faroe Islands as compared to the extract from Grenå, Denmark (Table 1). Different harvest times and origins are likely to lead to different chemical compositions (Sharma et al., 2018). The activity of the organic extracts (hexane and DCM:MeOH) was higher as compared to the MeOH:H_2_O extract in all seaweeds except *A. nodosum*, which generally showed the lowest activity. The only samples that showed a significant increase in activity of the fermented sample compared to the non-fermented sample were DCM:MeOH extracts of *S. latissima* from the Faroe Islands (*p* = 0.002), MeOH:H_2_O extracts of *S. latissima* from Grenå (*p* = 0.0003), hexane extracts of *L. digitata* from Grenå (*p* = 0.0003), MeOH:H_2_O extracts *P. palmata* from Grenå (*p* = 0.045) (Table1).

**TABLE 1:** Crude extracts. Extracted with: DCM, Dichloromethane; MeOH, methanol; H_2_O, Water. *Saccharina latissima* from Faroe Island and *Ascophyllum nodosum* harvested MAR-MAY were provided by Fermentation experts. All other seaweeds were provided by Nordic Seaweed. Mortality rate of extract exposed larvae are shown as mean ± SD. The mortality rate of *Ascaris suum* is for third stage larvae and first stage larvae for *Teladorsagia circumcincta.* The extracts were tested at a concentration of 1 mg/ml in both assays. N.D. = not done. Same superscripts indicate significant differences between the non-fermented and the corresponding fermented extracts (a: *p* < 0.05; b: *p* < 0.01; c: *p* < 0.001).

Species	Origin	Time of harvest	Fermentation	Extraction		Mortality rate (%) of	
				Protocol	Partition	*A. suum*	*T. circumcincta*
*Saccharina latissima*	Faroe Islands (cultured)	AUG 2016	−	Hexane		100 ± 0	100 ± 0
				Biphasic	DCM:MeOH	42.1 ± 4.5^b^	97.9 ± 1.8^a^
				Biphasic	MeOH:H_2_O	40.5 ± 12.2	99.3 + 0.9
			+	Hexane		100 ± 0	99.6 ± 0.7
				Biphasic	DCM:MeOH	100 ± 0^b^	99.7 ± 0.5^a^
				Biphasic	MeOH:H_2_O	18.7 ± 12.3	99.6 ± 0.7
	Grenå, Denmark (wild)	DEC 2017	−	Hexane		93.1 ± 2.7	N.D.
				Biphasic	DCM:MeOH	97.8 ± 2.1	N.D.
				Biphasic	MeOH:H_2_O	5.7 ± 3.0^c^	N.D.
			+	Hexane		87.3 ± 9.2	N.D.
				Biphasic	DCM:MeOH	85.3 ± 17.4	N.D.
				Biphasic	MeOH:H_2_O	62.2 ± 7.8^c^	N.D.
*Ascophyllum nodosum*	Norway (wild)	MAR-MAY 2017	−	Hexane		12.0 ± 6.0	6.4 ± 6.7
				Biphasic	DCM:MeOH	17.5 ± 4.2	−1.8 ± 1.6
				Biphasic	MeOH:H_2_O	22.8 ± 12.1	4.6 ± 3.6
			+	Hexane		10.7 ± 3.8	2.0 ± 2.6
				Biphasic	DCM:MeOH	4.3 ± 6.6	0.5 ± 2.4
				Biphasic	MeOH:H_2_O	35.8 ± 11.7	2.7 ± 1.6
	Bergen, Norway (cultured)	OCT 2017	−	Hexane		17.2 ± 6.7	N.D.
				Biphasic	DCM:MeOH	13.7 ± 8.2	N.D.
				Biphasic	MeOH:H_2_O	5.1 ± 10.7	N.D.
			+	Hexane		7.4 ± 4.5	N.D.
				Biphasic	DCM:MeOH	6.8 ± 6.3	N.D.
				Biphasic	MeOH:H_2_O	6.2 ± 4.7	N.D.
*Palmaria palmata*	Grenå, Denmark (wild)	DEC 2017	−	Hexane		91.9 ± 7.9	97.9 ± 3.2^b^
				Biphasic	DCM:MeOH	16.0 ± 12.0	2.9 ± 5.0^a^
				Biphasic	MeOH:H_2_O	21.9 ± 6.2^a^	44.0 ± 29.3^b^
			+	Hexane		72.3 ± 10.4	47.7 ± 32.6^b^
				Biphasic	DCM:MeOH	30.7 ± 1.9	53.7 ± 50.9^a^
				Biphasic	MeOH:H_2_O	40.6 ± 9.4^a^	98.6 ± 1.5^b^
*Laminaria digitata*	Grenå, Denmark (wild)	DEC 2017	−	Hexane		46.5 ± 2.9^c^	15.8 ± 4.4^c^
				Biphasic	DCM:MeOH	94.7 ± 2.8	87.3 ± 12.4^a^
				Biphasic	MeOH:H_2_O	14.5 ± 3.5	14.3 ± 17.4^c^
			+	Hexane		86.0 ± 5.3^c^	98.0 ± 1.6^c^
				Biphasic	DCM:MeOH	90.7 ± 8.1	99.7 ± 0.8^a^
				Biphasic	MeOH:H_2_O	24.6 ± 8.6	98.1 ± 1.3^c^

To confirm that the activity was not only limited to one parasite species, the crude extracts were also tested against first stage larvae (L1) of *T. circumcincta*, a GI nematode ubiquitous in sheep production systems (Venturina et al., 2013). A similar pattern in activity was observed in relation to the potency of the extracts. A mortality of >97% was induced by all extracts from *S. latissima*, all extracts from fermented *L. digitata*, the MeOH:H_2_O from fermented *P. palmata* and hexane extract from non-fermented *P. palmata*. The DCM:MeOH extract from non-fermented *L. digitata* induced a mortality of >87% (Table 1). The remaining extracts from *P. palmata* induced nil to moderate mortality with high variation and, consistent with the *A. suum* data, extracts from *A. nodosum* induced only very minor mortality. Significant increases in activity between fermented compared to non-fermented samples were observed in 5 cases out of 12; hexane extracts from *L. digitata* (*p* < 0.0001), DCM:MeOH extraction from *L. digitata* (*p* = 0.03) and *P. palmata* (*p* = 0.015)*,* MeOH:H_2_O extraction from *L. digitata* (*p* < 0.0001) and *P. palmata* (*p* = 0.006). A statistically significant increase in activity was also observed for the fermented DCM:MeOH extraction of *S. latissima* as compared to non-fermented, however, both samples still exhibited activity above 97% (*p* = 0.046). In contrast, hexane extracts of *P. palmata* showed a significant decrease in activity between the non-fermented and the fermented samples (*p* = 0.002).

**FIGURE 1:**
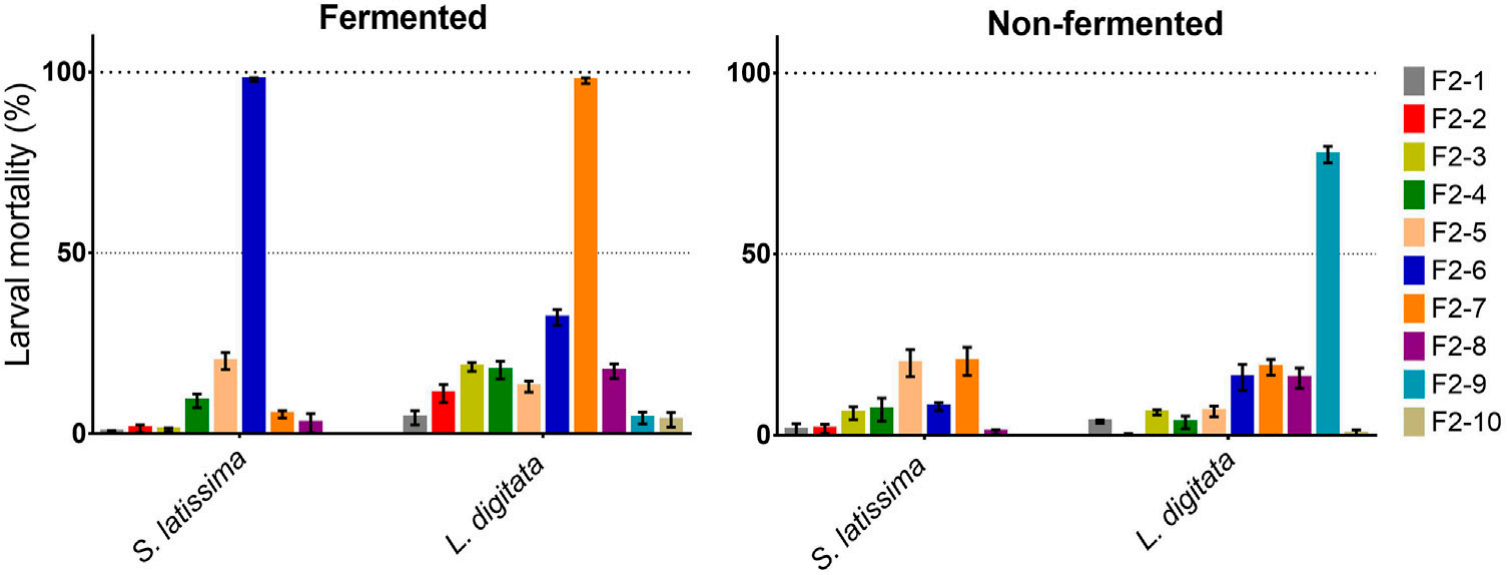
Larval mortality of *Ascaris suum* third stage larvae by subfractions of fraction F1-2 from fermented and non-fermented *Saccharina latissima* (F2-1 to -8) and *Laminaria digitata* (F2-1 to -10). The results are a mean of two experiments each performed in duplicates and counted after 24 h incubation. Error bars represent SEM. Samples tested at 100 μg/ml.

Based on the two parasite assays, two of the selected seaweed species (*S. latissima*, *L. digitata*) appeared to contain compounds with broad-spectrum anti-parasitic activity, against both clade III and V nematodes (Blaxter et al., 1998) with more than 80% mortality in most extracts at a concentration of 1 mg/ml. The DCM:MeOH extracts of both fermented and non-fermented S. *latissima* and *L. digitata* were chosen for further investigation and bio-guided fractionation via flash chromatography. DCM:MeOH fractions were prepared by firstly extracting with DCM:MeOH (2:1), followed by flash chromatography over a gradient and finally analysis by LC-MS/MS. Fractions from both *S. latissima* and *L. digitata* showed activity against *A. suum* L3 over several fractions after 24 h at a concentration of 500 μg/ml (Table 2). For both species, fractions F1-1 to 1-3 were identified as the overall most biologically active. Fraction F1-2 displayed the highest larval mortality in all cases, and was therefore sub-fractionated to yield a further eight (*S. latissima*) or ten (*L. digitata*) subfractions (Figure 1). For fermented *S. latissima,* subfraction F2-6 was significantly more active than all other subfractions (>95% larval mortality against *A. suum*), while subfractions F2-7 of fermented and subfraction F2-9 of non-fermented *L. digitata* were also highly active. The subfractions showed differences in activity between the fermented samples and the non-fermented samples. For *S. latissima*, mortality in the subfraction F2-6 was significantly higher in the fermented than non-fermented subfraction (*P* = <0.0001), but for fraction F2-3 and F2-7 activity was higher in the non-fermented fraction (*p* = 0.03, *p* = 0.01). For *L. digitata* the mortality was significantly higher in subfractions F2-2 to F2-7 for fermented samples as compared to corresponding non-fermented samples, but significantly lower in F2-9 (*P* = <0.0001).

**TABLE 2:** Mass and anti-parasitic effects of isolated seaweed fractions. Tested for antiparasitic activity against *Ascaris suum* third stage larvae at 500 μg/ml after 24 h incubation, the results represent mean ± SD of duplicates from two different experiments.

	Non-fermented fractions (Non-FER)					Fermented fractions (FER)				
	F1-1	F1-2	F1-3	F1-4	F1-5	F1-1	F1-2	F1-3	F1-4	F1-5
*Saccharina latissima* ^a^										
Mass (mg)	107	815	58	50	1,079	158	905	182	280	1,664
Proportion of crude extract (%)	5	39	3	2	51	5	28	6	9	52
Larval mortality (%)	67.1 ± 21.0	95.2 ± 4.9	47.9 ± 25.7	28.1 ± 20.1	4.3 ± 5.5	95.6 ± 3.8	99.7 ± 0.6	47.9 ± 16.1	25.8 ± 2.1	7.8 ± 4.3
*Laminaria digitata* ^b^										
Mass (mg)	489	1,187	160	132	1,586	505	3,721	387	226	830
Proportion of crude extract (%)	14	33	4	4	45	9	66	7	4	15
Larval mortality (%)	20.7 ± 16.6	89.1 ± 11.1	82.7 ± 6.2	25.5 ± 14.4	4.6 ± 5.7	87.2 ± 0.9	94.0 ± 5.9	68.5 ± 11.8	64.3 ± 17.6	10.8 ± 4.0

a Faroe Islands.

b Denmark.

**FIGURE 2:**
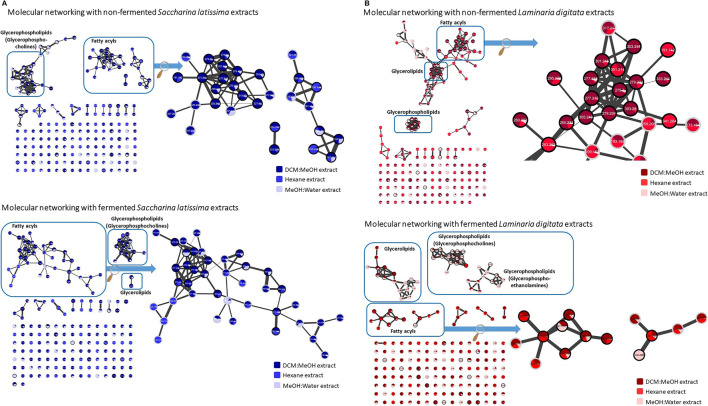
**(A)** Molecular networking of seaweed extracts using MS/MS data. The entire networks of non-fermented and fermented *Saccharina latissima* from Faroe Islands and isolated compounds within molecular category. **Spectra node legend**: Bold edges: putatively identification by spectral library matching. Numbers: precursor ion (m/z). Colors: distribution of spectra per sample. **(B)** Molecular networking of seaweed extracts using MS/MS data. The entire network of non-fermented and fermented *Laminaria digitata* and isolated compounds within molecular category. **Spectra node legend**: Bold edges: putatively identification by spectral library matching. Numbers: precursor ion (m/z). Colors: distribution of spectra per sample.

Using bio-guided fractionation and reverse-phase HPLC purification we subsequently isolated five compounds (**1**–**5)** from subfraction *S. latissima* FER-F2-6 and four compounds (**1**–**3**+**6)** from subfraction *L. digitata* FER-F2-7. Using MS/MS data (Supplementary material Image 2), MS-FINDER, and GNPS molecular networking (Figures 2A,B) all isolated compounds were found to be fatty acids or derivatives thereof. Based on their molecular formula, retention time (Table 3) and MS/MS fragmentation pattern (Supplementary material Image 2) their were tentatively identified as C18:4 (1), C20:5 (2), C18:3 (3), C22:6 (4), C20:4 (5) and Monoacylglycerol (MG 20:5) (6). Detailed formula and structure information of other candidates of compound 1-6 is available in Supplementary material Data Sheet 1. The tentative identification led to the following compounds Stearidonic acid (**1**), Eicosapentaenoic acid (**2**), Alpha-Linolenic acid (**3**), Docosahexaenoic acid (**4**), Arachidonic acid (**5**), and MG 20:5 (**6**) since they achieved the highest scores in MS-FINDER (Supplementary material Data Sheet 1). Compound **1-5** was isolated in very high amounts during the purification. The structures have been found as the major lipids in the two seaweed species, and our findings are thus in line with previous discoveries (Foseid et al., 2020; Monteiro et al., 2020).

**TABLE 3:** Spectral data of the six isolated compounds.

Compound	Retention time	Annotation	Formula	Theoretical m/z	Experimental m/z
				[M + H]+	[M + H]+
1	9.05	Stearidonic acid	C_18_H_28_O_2_	277.2162	277.2164 (0.72 ppm)
2	9.42	Eicosapentaenoic acid	C_20_H_30_O_2_	303.2319	303.2323 (1.32 ppm)
3	9.50	Alpha-linolenic acid	C_18_H_30_O_2_	279.2319	279.2318 (0.36 ppm)
4	9.76	Docosahexaenoic acid	C_22_H_32_O_2_	329.2475	329.2477 (0.61 ppm)
5	9.92	Arachidonic acid	C_20_H_32_O_2_	305.2475	305.2480 (1.64 ppm)
6	8.86	MG 20:5	C_23_H_36_O_4_	377.2686,399.2506 [M + Na]+	377.2686 (0.00 ppm) 399.2510 (1.00 ppm)

All six compounds were tested individually against *A. suum* L3 and found to hold little to moderate activity at a concentration of 50 μg/ml Compounds **4** and **5** displayed <6% mean larval mortality, and compound **1**, **2**, **3**, and **6** had a mean activity of 31, 47, 63, and 14%, respectively. EC_50_ values were calculated to be 82.3 (CI 95%: 76.4–88.3) µg/ml for **1**, 53.9 (47.6–61.0) µg/ml for **2** and 41.1 (34.6–48.6) µg/ml for **3**.

**FIGURE 3:**
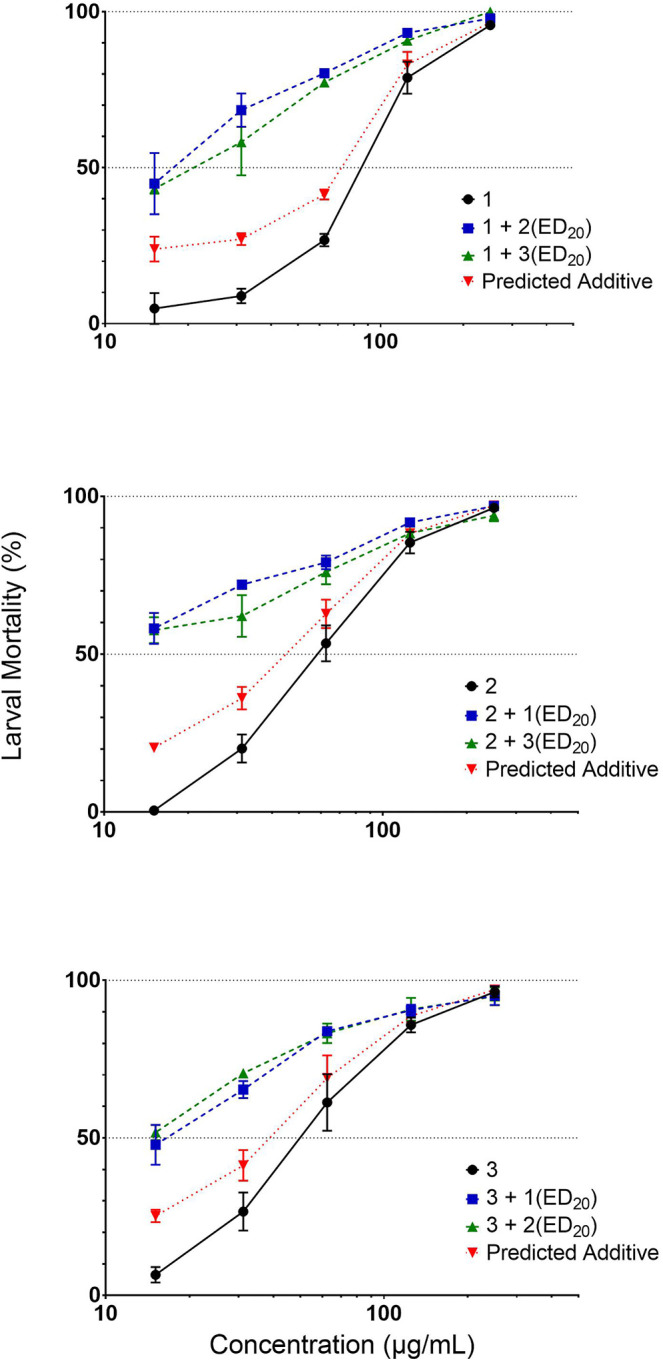
Synergistic effects of isolated compounds *in vitro*. Percentage mortality of *Ascaris suum* third stage larvae in the presence of an active isolated compound (**1**–**3**) with or without EC_20_ concentration of either of the two other active compound. Results are the mean (±SEM) of triplicates.

The highly active subfraction *L. digitata* non-FER-F2-9 contained compounds **1**, **2** and **3**, with **5** and **6** in lesser amounts. The *S. latissima* non-FER-F2-6 subfraction with low larval mortality (7.9 ± 2.1%) at 100 μg/ml was also found to contain compounds **1**, **2**, **5** and **6**, while the most potent compound **3** was only present at trace levels. This may explain the low activity compared to the corresponding fermented sub-fraction (Figure 1). The moderate activity of the individual compounds, compared to the potency of the fraction they were derived from, suggests a synergistic effect of the mixture of the compounds. Thus, the three most active compounds were tested for synergistic effects by quantifying activity of one compound at several different concentrations combined with a fixed concentration (corresponding to the EC_20_) of the two other compounds. The predicted activity of the compounds was calculated based on the assumption of them having an additive effect, with activity higher than these predicted values defined as synergy (Williams et al., 2012). This approach clearly indicated that activity in the fractions was due to synergy between the different active compounds, since an increase in activity above the predicted values was observed for all combinations (Figure 3), especially at lower concentrations.

Overall, our results show that the Nordic seaweeds *S. latissima* and *L. digitata* contain C18–20 poly-unsaturated fatty acids (PUFAs) with *in vitro* effects against nematode larvae. Furthermore, our results confirm that even though these compounds individually only have moderate effects, the synergistic effects enable a potent anti-parasitic effect. Fermentation did change the molecular network of the seaweeds and activity of different subfractions, but the same active compounds were present in both fermented and non-fermented active samples. Although similar in their basic structures, not all isolated compounds displayed anti-parasitic activity. Capon et al. (1998) observed that oxidation or reduction of the terminal double bond substantially diminished the anti-parasitic activity of the seaweed compound *trans*-dihydroxytetrahydrofuran and its derivatives (Capon et al., 1998).

Limited research has been performed on direct effect of fatty acids on helminths. The PUFA linoleic acid has shown nematocidal activity against the nematode *Caenorhabditis elegans* (Stadler et al., 1994; Panda et al., 2020), but also against *H. contortus in vitro* with a EC_90_ of 0.93 mg/ml (Ayers et al., 2010). One study found a mix of fatty acids (unsaturated and saturated) at concentrations >5.8 mg/ml showed high mortality (>90%) of *H. contortus* L3 *in vitro* (Pineda-Alegría et al., 2020). Our study showed a high mortality (>90%) at concentrations of only 0.25 mg/ml for all our isolated compounds against *A. suum* and >90% mortality for all dual combinations at 0.13 mg/ml except one (**2** + **3** (EC_20_)). It is possible that the PUFAs isolated from seaweed hold higher bioactive properties than commercially available acids given the more than 10-fold higher activity at a lower dose in our study, although it should be kept in mind that the experiments were performed on different parasites. Fatty acids constitute important building blocks of cell membranes and take part in regulation of membrane fluidity (De Carvalho and Caramujo, 2018). It may be hypothesized that exposure to the fatty acids identified in the current study could interact with and compromise the integrity of the cuticle causing lethal damage to the parasites. *In vitro* exposure of the trematode, *Schistosoma* spp. to arachidonic acid above 20 μM also led to disintegration of surface membranes and eventually parasite death (Tallima et al., 2005). In addition, the effects of linoleic acid on *C. elegans* was speculated to affect ionotropic neurotransmitter receptors, a target of some common anthelmintics (Panda et al., 2020).

To the best of our knowledge only one study on the effects of fatty acid effects on helminths have been performed *in vivo*. Administration of 25 g/day dietary PUFA (fish oil, n-3 omega) in calves infected with *Ostertagia ostertagi* and *Cooperia oncophora* did not significantly affect the number of worms, although the worm burdens were 28% lower than in infected controls for intestinal worms (Muturi et al., 2005).

In conclusion, the seaweeds *L. digitata* and *S. latissima* have potential as a source of anti-parasitic compounds or rather, as bioactive feeds (or feed ingredients) against parasites in livestock, and our study show fatty acids to be among the active compounds. Further studies will reveal if the *in vitro* properties of seaweed against helminths translate to *in vivo* effects in infected animals fed diets with a seaweed component and if so which mechanisms are involved.

## Experimental Section

### Macroalgae Material

The samples originated from four different species of Macroalgae (seaweed): *Palmaria palmata*, *Laminaria digitata*, *Saccharina latissima* and *Ascophyllum nodosum* and were supplied by the companies Nordic Seaweed ApS (NS) and Fermentation experts A/S (FEX). The seaweeds originated from Denmark, Faroe Islands and Norway, place of origin and time of harvest are listed in Table 1. Samples from Grenå, Denmark, were wild growing and collected at coordinates: 56°26′43.07″N, 10°57′28.33″E and 56°26′28.33″N, 10°57′29.50″E. *S. latissima* from Faroe Islands and *A. nodosum* from Bergen, Norway were cultured. The samples were supplied as dried and milled material. Both fermented and non-fermented material (*N* = 12) was supplied for each seaweed species. Material from Nordic Seaweed ApS was fermented aerobically by "*Lactobacillus plantarum* NT" for 20–24 h and dried indoor by wind turbines at 30–35°C. Material from Fermentationexperts A/S was fermented using a two-step anaerobic solid state fermentation process with an in-house bacterial mix inoculum (*Lactobacillus* spp.), and then dried in a spin flash dryer as described elsewhere (Satessa et al., 2020).

### Crude Extractions of Seaweeds

Seaweed samples were extracted with two protocols leading to three extracts, using a similar approach as previously published (Peña-Espinoza et al., 2020). For the hexane extraction, 30 g of seaweed was extracted three times at room temperature, with 100 ml of hexane and 10 min sonication. The extracts were filtered on filter paper and solvents were removed under reduced pressure leading to the organic extract. For the DCM:MeOH biphasic extraction, 30 g of seaweed sample was extracted at room temperature. First 200 ml of MeOH:H_2_O (1:1) was added and sonicated for 10 min, followed by addition of 150 ml of a mixture of DCM:MeOH (2:1) to the mixture and sonicated for additional 10 min. After filtration on filter paper, the two phases (organic and hydroalcoholic) were separated and the solvents evaporated under reduced pressure, resulting in two extracts. The hydroalcoholic extracts (partition MeOH:H_2_O) were desalted on SPE cartridges (Waters Sep-Pak C18 20 cc Vac Cartridge, 5 g, Ref: WAT036925) with 10 ml H_2_O to remove salts, followed by elution with 15 ml of MeOH to recover the desalted extracts. An overview of all samples and resulting extracts can be seen in Table 1. All extracts were analyzed on LC-MS and tested for anti-parasitic activity.

### Purification of Seaweed Fatty Acids

Based on the biological activities against parasites, four samples were chosen for further extraction and fractionation (Table 2). Each sample was extracted at room temperature with 600 ml of a DCM:MeOH (2:1) mixture and sonicated for 10 min. After filtration, the extraction was repeated twice with 200 ml DCM:MeOH (2:1) and solvents were removed under reduced pressure. The extract was solubilized in MeOH/DCM, and a Silica-diol phase was added before solvent evaporation using a rotary evaporator. The extract was then submitted to flash chromatography using a 100 g snap cartridge containing diol-functionalized silica (Flow: 50 ml/min) with the following gradient: 1) Hexane 100% during 2 min, 2) Hexane/Isopropanol: 0–100% Isopropanol over 23 min, 3) 100% Isopropanol held for 2 min, 4) Isopropanol/water: 0–100% water over 3 min and 5) Water 100% during 2 min. The cartridge was previously equilibrated with hexane for 3 min. For each extract, 16 fractions (100 ml each) were collected and grouped into five fractions and the solvents evaporated using rotary evaporation. The dried fractions were subsequently used for anti-parasitic assays.

Fraction 1–2 (Table 2) was selected for further fractionation on flash chromatography using a Biotage Cartridge Snap KP-C18-HS 30 g (Ref: FSL0-1,118–0,030), a H_2_O/MeOH solvent system and a 25 ml/min flow. The dry deposit was realized by mixing 1.8 g C18 silica with the extract. The following gradient was employed: 1) MeOH 30% held for 2 min, 2) MeOH 30–100% over 33 min, 3) MeOH 100% held for 5 min (Equilibration time: 3 min). A total of 36 tubes (30 ml) were collected and grouped into 8 fractions (Sl (FER)-F2-1 to 2–8) for fermented *S. latissima,* and 10 fractions (Ld (FER)-F2-1 to 2–10) for fermented *L. digitata*. Fraction Sl (FER)-F2-6 was then fractionated again on flash chromatography using the same cartridge, H_2_O/MeOH solvent system and a 25 ml/min flow with the gradient: 1) MeOH 70% held for 2 min, 2) MeOH 70–100% over 20 min, 3) MeOH 100% held for 5 min (Equilibration time: 3 min). Nine fractions were collected (Sl (FER)-F2-6-1 to Sl (FER)-F2-6–9). Fraction Sl (FER)-F2-6-6, Sl-F2-6-7 and Sl (FER)-F2-6-8 were grouped into a single fraction which was subjected to reverse-phase HPLC purification (Phenomenex, Luna C18 (2) 100 Å 250 × 10 mm, 5 µm) using an isocratic elution with 79% H_2_O-CH_3_CN (+0.1% formic acid) at a flow rate of 5 ml/min. Fraction Sl (FER)-F2-6-6 to -8 gave compound **1** (4 mg, RT = 17.5 min), **2** (11 mg, RT = 20 min), **3** (3 mg, RT = 21 min), **4** (19 mg, RT = 17 min) and **5** (14 mg, RT = 19 min). Fraction Ld (FER)-F2-7 was subjected to the same reverse-phase HPLC. The Ld (FER)-F2-7 fraction gave compound **1** (10 mg, RT = 17.5 min), **2** (25 mg, RT = 20 min), **3** (28 mg, RT = 21 min) and **6** (9 mg, RT = 14 min).

### Liquid Chromatography - Mass Spectrometry (LC-MS) Analysis

All extracts were solubilized at concentration of 1 mg/ml, in isopropanol for hexane extracts or MeOH for the others extract. Pure compounds were prepared at a concentration of 0.2 mg/ml in MeOH. Ultra-high Performance Liquid Chromatography-High Resolution Mass Spectrometry (UHPLC-HRMS) was performed on a Dionex Ultimate 3000 RS equipped with a diode array detector and coupled to Bruker QTOF Maxis with an electrospray ionization source. The analyses (injection volume: 2 µL) were performed on a reversed-phase column (Phenomenex Luna Omega C-18, 150 × 2.1 mm, 1.6 µm) employing a gradient of 10–100% CH_3_CN in water over 10 min followed by 3 min at 100% CH_3_CN (all solvents buffered with 0.1% formic acid) with a flow rate of 0.5 ml/min. Analyses were performed in positive mode.

### Molecular Networking

Molecular networking of the compounds present in the crude extracts of *S. latissima* and *L. digitata* (fermented and non-fermented samples) was performed using the online platform of the Global Natural Product Social Molecular Networking (GNPS) web platform (https://gnps.ucsd.edu). First, LC-MS chromatograms of the three crude extracts from each seaweed species (hexane, DCM:MeOH and MeOH:H_2_0 extracts) were converted to mzXML files and pre-processed (by the same seaweed species and fermentation status) with Mzmine (Pluskal et al., 2010) using the following parameters: 1) Mass detection: MS level 1; retention time 1–12 min; noise level 1,000. MS level 2; retention time 1–12 min; noise level 300. 2) Chromatogram builder: retention time 1–12 min; MS level 1; min time span 0.01 min; min height 3,000; m/z tolerance 0.01 m/z or 30.0 ppm 3) Chromatogram deconvolution: baseline cut-off min peak height 2000; peak duration 0.01–3.00; baseline level 800; m/z range for MS2 scan pairing 0.02; retention time range for MS2 scan pairing 0.3 min 4) Isotopic peak grouper: m/z tolerance 0.0 m/z or 20.0 ppm; retention time tolerance 0.2 min 5) Join aligner: m/z tolerance 0.0 m/z or 30.0 ppm.; weight for m/z 75; retention time tolerance 0.5 min; weight for retention time 25. 6) Gap filling: intensity tolerance 10.0%; m/z tolerance 0.01 m/z or 30.0 ppm; retention time tolerance 0.5 min. Data was further subjected to filtering of MS/MS peaks (peak list row filter) selecting the following settings: minimum peaks in a row 2; minimum peaks in an isotope pattern 2; keep only peaks with MS2 scan (GNPS). Resulting feature table with aligned peaks (.csv) and consensus MS2 spectra (.mgf) files were exported for further analysis in GNPS.

Classical molecular networks were created using the online workflow (https://ccms-ucsd.github.io/GNPSDocumentation/) on the GNPS website (http://gnps.ucsd.edu), as previously described (Wang et al., 2016). The data was filtered by removing all MS/MS fragment ions within ± 17 Da of the precursor m/z. MS/MS spectra were window filtered by choosing only the top 6 fragment ions in the ± 50 Da window throughout the spectrum. The precursor ion mass tolerance was set to 0.04 Da and a MS/MS fragment ion tolerance of 0.04 Da. A network was then created where edges were filtered to have a cosine score above 0.6 and more than 6 matched peaks. Further, edges between two nodes were kept in the network if and only if each of the nodes appeared in each other’s respective top 10 most similar nodes. Finally, the maximum size of a molecular family was set to 100, and the lowest scoring edges were removed from molecular families until the molecular family size was below this threshold. The library spectra were filtered in the same manner as the input data. All matches kept between network spectra and library spectra were required to have a score above 0.7 and at least 5 matched peaks. The mass spectral molecular networking are accessible here: *S. latissima* extracts (non-fermented samples): https://gnps.ucsd.edu/ProteoSAFe/status.jsp?task=47f091c230c440a5b909bafde6456160; *S. latissima* extracts (fermented samples): http://gnps.ucsd.edu/ProteoSAFe/status.jsp?task=610990c014c9403c97dba218e59f8b35; *L. digitata* extracts (non-fermented samples): http://gnps.ucsd.edu/ProteoSAFe/status.jsp?task=a3ab8245b68a42a29dbf2b98f8949b69; *L. digitata* extracts (fermented sample: https://gnps.ucsd.edu/ProteoSAFe/status.jsp?task=e4862ede88fd4ab18a00ab4da747ab89.

### Compound Identification

The raw data was first converted to Abf format (Analysis base File Converter v. 4.0.0) and then imported to MS-DIAL (version 4.24) (Tsugawa et al., 2020). The detailed description of peak peaking, deconvolution and alignment is available in Supplementary material Data Sheet 1. Then, the results were exported to MS-FINDER (Version 3.50) (Lai et al., 2018) for tentative identification. MS/MS spectrums of compound 1-6 were searched on several public databases and compared with *in silico* MS/MS spectrums generated in MS-FINDER. Candidates with highest scores were chosen.

### Parasites

Gravid *A. suum* were collected from pig intestines at the local slaughterhouse. Eggs were isolated from dissected uteri and embryonated for two months at room temperature (22°C) (Oksanen et al., 1990). Once a week cultures were aerated. After this period, they were stored at 4°C in 1 M H_2_SO_4_ at a concentration of ∼25,000 eggs/mL until usage shortly after. To obtain L3, embryonated eggs were washed in Hank’s Balanced Salt Solution (HBSS) and larvae hatched by stirring with 2 mm glass beads for 40 min at 37°C in a 5% CO_2_ incubator. The L3 were allowed to migrate overnight at 37°C (5% CO_2_) from a 20 µm sieve into sterile HBSS (supplemented with 100 U/mL penicillin, 100 μg/ml streptomycin and 0.25 μg/ml amphotericin B) in a conical sedimentation glass. The larvae were collected from the bottom of the glass and suspended in heated larval culture media (37°C RPMI 1640 supplemented with 2 mM l-glutamine, 100 U/mL penicillin and 100 μg/ml streptomycin).

The *T. circumcincta* assay was performed essentially as described previously (Escala et al., 2020). Briefly, eggs were collected from feces from two 3-months old Merino lambs experimentally infected with a field strain of *T. circumcincta* (susceptible to anthelmintics) maintained in the laboratory (Martínez-Valladares et al., 2012). The infection was approved by the Animal Ethics Committees of the Instituto de Ganadería de Montaña, Spanish Council of Research (CSIC) and Junta de Castilla y León, following national regulations (R.D. 53/2013). The freshly collected feces were washed under tap water using three sieves (placed in descending order: 150, 80 and 32 µm). The sediment on the 32 µM sieve was collected and then washed by centrifugation two times, first with water and afterward with Saturated Salt Solution. Afterward, the eggs were collected, washed with distilled water and placed in a sedimentation glass to incubate for 24 h at 23°C. The next day the first stage larvae (L1) were recovered from the bottom of the glass and concentrated at ∼1.0 larvae/µL in distilled water.

### Larval Mortality Assay

The *A. suum* assay was performed using flat bottom 96 well-plates, in which 1.5 µL of extract or fraction at an appropriate concentration (see below) dissolved in DMSO were added to 150 µL of larvae mixture (∼100 larvae) in triplicates and incubated at 37°C. DMSO (1%) was used as a negative control. Moving or coiled-up larvae were counted as alive, and immobile and straight larvae as dead. The survival percentage of each replicate was calculated ((alive/(alive+dead))x100), and results corrected according to the negative controls to get the mortality rate, using the following calculation:

$$\text{Mortality Rate}=\;\left(1-\frac{\text{survival \% of larvae exposed to extract}}{\text{survival \% of negative controls}}\right)$$

Crude extracts from the four seaweed species were tested in triplicates at 1 mg/ml at 24, 48 and 72 h incubation, while fractions were tested as duplicates on two plates and read after 24h at 500 μg/ml and 100 μg/ml, respectively. Pure compounds were tested as duplicates on four plates at 50 μg/ml. We calculated the EC_50_ of the three isolated compounds that were selected based on an experiment performed in triplicate, using a twofold dilution of concentration from 250 μg/ml to 7.8 μg/ml of the active compounds. The same design was used for testing the synergy between the three compounds, using the same concentrations but ranging from 250 μg/ml to 15.1 μg/ml and adding the equivalent of EC_20_ of the separate compounds to each dilution row. EC_20_ calculation were based on previous EC_50_ curves and found to be 63 μg/ml for compound **1**, 28 μg/ml for compound **2**, and 20 μg/ml of compound **3** using the equation:

$${\text{EC}}_{20}={\left(\frac{20}{100-20}\right)}^{1/\text{Hill Slope}}*{\text{EC}}_{50}$$

Compounds were said to display synergy if the mortality of *A. suum* larvae were above the predictive additive effect calculated using the equation:

$$\text{Predicted addivitve effect}=\left(1-\frac{\text{mortality of }Ascaris\;suum\;\text{L}3\text{ exposed to Compound X }}{100}\right)\text{*}\left(1-\frac{{\text{EC}}_{20}\text{ of compound Y}}{100}\right)\text{*}100$$

For the *T. circumcincta* (L1) assay the plate was set up as described for the *A. suum* (L3) assay. Extracts were screened at a concentration of 1 mg/ml and 1% DMSO in triplicates in 96 well-plates on 2 adjacent days with different larval batches. The anthelmintic Ivermectin (Oramec 8%, Boehringer Ingelheim Animal Health España) at 100 µM was used as a positive control and DMSO 1% was used as a negative control. The assay was counted after 48 h incubation at 23°C, and the mortality rate was calculated as for *A. suum*.

### Statistical Analysis

Prism 7.04 was used for statistical analysis of mean and standard deviation and calculation of EC_50_ of isolated compounds by non-linear regression. Depending on the distribution and variance of the replicas, *t*-test, Welsh's *t*-test or Mann-Whitney test was used to compare the activity of the fermented sample to the corresponding non-fermented sample of the crude extracts as well as F2 corresponding fractions.

## Data Availability

The original contributions presented in the study are included in the article/Supplementary Material, further inquiries can be directed to the corresponding author.
