# Supplementary figures and images for: Bio-Guided Fractionation and Molecular Networking Reveal Fatty Acids to Be Principal Anti-Parasitic Compounds in Nordic Seaweeds
Source: Front Pharmacol. 2021 Jun 2;12:674520. doi: 10.3389/fphar.2021.674520 (PMC8206555; doi:10.3389/fphar.2021.674520)

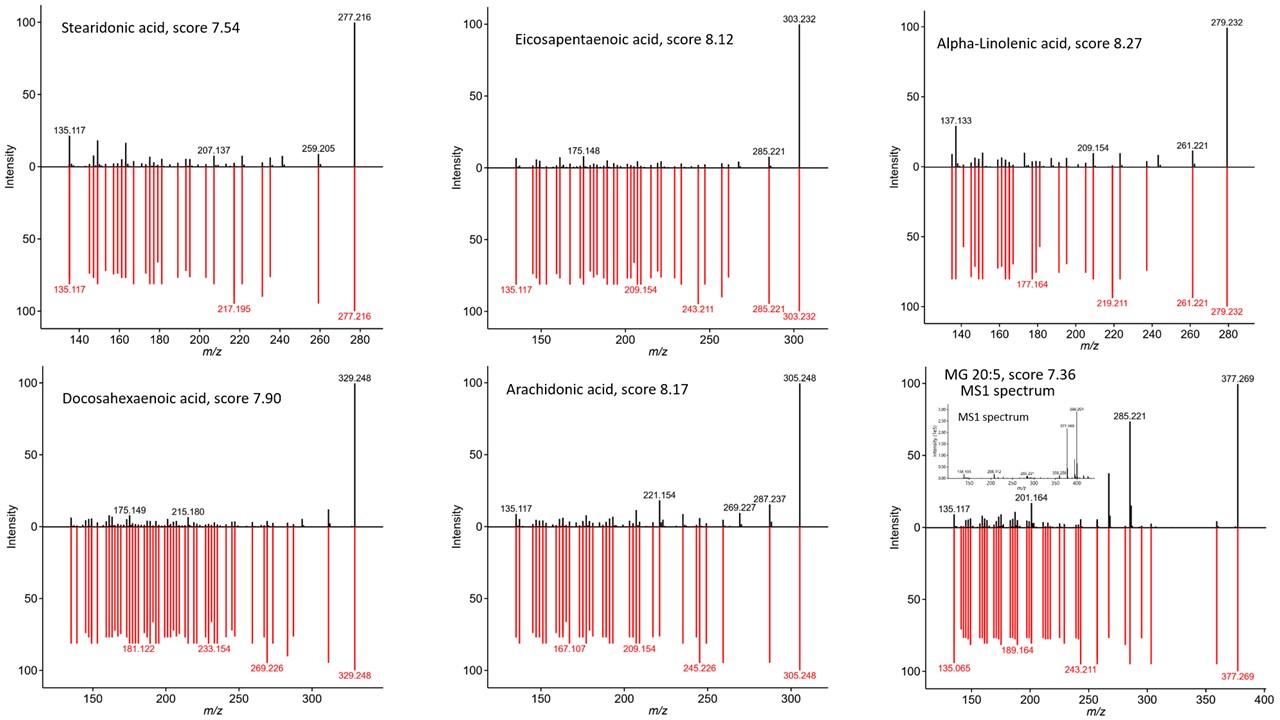

Supplement: Supplementary file 1 [file Image2.jpg]

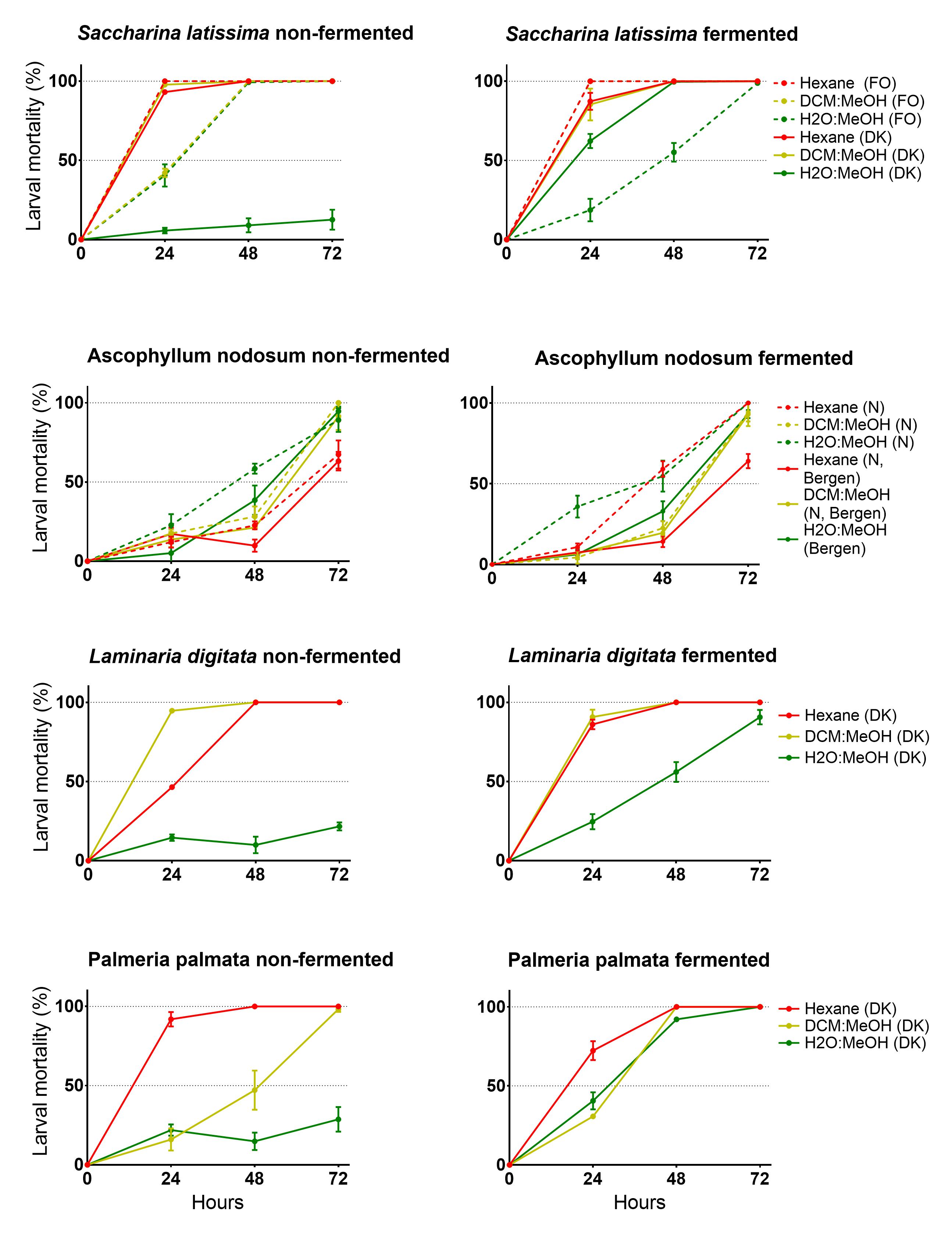

Supplement: Supplementary file 2 [file Image1.JPEG]
